# Riding Pontic: A Tool to Keep Patients Smiling

**DOI:** 10.5005/jp-journals-10005-1204

**Published:** 2013-08-26

**Authors:** Narendra Shriram Sharma

**Affiliations:** Assistant Professor, Department of Orthodontia, Sharad Pawar Dental College, Wardha, Maharashtra, India, e-mail: sharmanarendra047@gmail.com

**Keywords:** Corrective orthodontics, Adult orthodontics, Esthetic

## Abstract

All patients expect a beautiful smile at the completion of orthodontic treatment, but some patients show concern regarding their appearance while undergoing treatment. The appearance of a gap from a missing tooth can be a concern, especially if it is in the display zone of a patient's smile. If the treatment plan includes prosthetic replacement of the missing tooth rather then space closure, then space maintenance is also an issue. In an appearance conscious patient use of riding pontic as space maintainers is a good option during treatment.

**How to cite this article:** Sharma NS. Riding Pontic: A Tool to Keep Patients Smiling. Int J Clin Pediatr Dent 2013;6(2): 127-131.

## INTRODUCTION

An expectation of a beautiful smile at the end of orthodontic treatment is a primary concern for all patients, but most are also concerned with appearance while undergoing treatment. This is evident in the attempts by manufacturers to meet the esthetic demand of patients while undergoing orthodontic treatment, including making metal brackets smaller, developing lingual or ‘invisible’ brackets, esthetic archwires, plastic brackets and translucent ceramic brackets.^3^

The demand for adult orthodontics is increasing. It is not unusual for orthodontic patients to have missing anterior teeth. In some patients, a hopeless anterior tooth was extracted prior to beginning orthodontic treatment. In some of these patients, orthodontic treatment alone may solve the problem. However, in others prosthetic replacement of missing teeth is the most suitable treatment. In such patients, preprosthetic orthodontic treatment often has to be performed to facilitate the restorative treatment by positioning the teeth for the best possible esthetic and functional results. It is important to maintain the esthetics of those individuals undergoing fixed orthodontic treatment. Riding pontics are a good solution for these patients.

## RIDING PONTIC

Riding pontics^2^ are temporary prostheses used during fixed orthodontic treatment in patients with missing teeth and can be used for any missing teeth. It is especially good when one or more anterior teeth are missing.

### Fabrication

The following are the steps for fabrication of riding pontics:

- *Color matching*: The color of the acrylic tooth should be matched to the color of adjacent natural tooth.
- *Mesiodistal width determination*: When a single anterior tooth is missing, mesiodistal width of the pontic should be determined by considering the width of the contralateral natural tooth. When teeth are missing bilaterally, the mesiodistal width of the pontic should be determined by analyzing the space available and the dimensions of the remaining natural teeth.
- *Height determination*: The cervical end of the pontic should touch the gingiva with a smooth contour. If the cervical end of the pontic does not touch to the gingiva, then the negative space between the pontic and the gingiva can affect the esthetics, especially in high smile line patients. The incisal edge or cusp tip of the pontic should be in harmony with the adjacent natural tooth for maximum esthetics.
- Bonding the bracket on the acrylic tooth.
- *Try in*: The pontic should be held in the edentulous space to preview the final outcome. Any final reshaping should be done to obtain the best esthetic result.
- *Ligation*: The pontic can be ligated to the archwire either by a ligature wire or elastic module. Tight ligation of pontic by ligature wire provides the best stability. Minimum play between the archwire and bracket slot is desirable.

### Benefits of using Riding Pontics

- Improvement of esthetics during orthodontic treatment.
- Development of abnormal habits such as tongue thrusting and defective speech can be prevented.
- Exact mesiodistal width of the missing tooth can be maintained.
- Midline matching along with riding pontic is easier when unilateral incisor is missing.
- Psychosocial status of the patient can be improved.

### Problems with Riding Pontics

Labiopalatal rotational control of the riding pontic is difficult with round archwire; however, movement can be limited with rectangular archwires. Bond failure of the pontic may occur during treatment.

## CASE REPORT

**Fig. 1:**
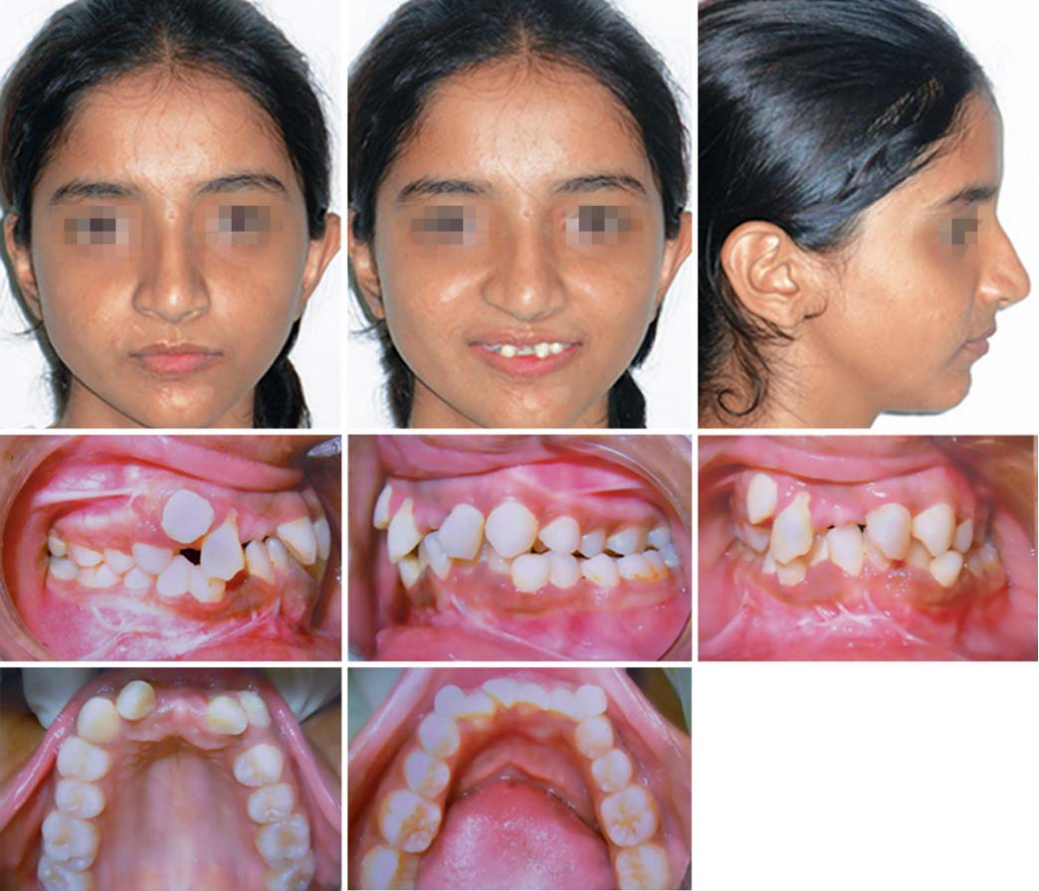
Extraoral and intraoral views

A 12-year-old female reported to the Department of Oral Surgery with chief complaint of fracture of jaw and missing both central incisors in road side accident. On examination it reveals that there is fracture of lower jaw in parasymphysis region, missing both central incisors, impacted both lower canine and crowing in both the jaw. She had no history of orthodontic treatment. She had a pleasing profile. Class I molar relationship with missing both maxillary central incisors and moderate crowding in the maxillary and mandibular anterior region (Fig. 1). Nonextraction treatment was planned. Treatment objectives were repair of fracture of lower jaw, regaining the space for prosthetic replacement of central incisors, relieving the crowding and maintaining the existing class I molar relationship.

**Fig. 2:**
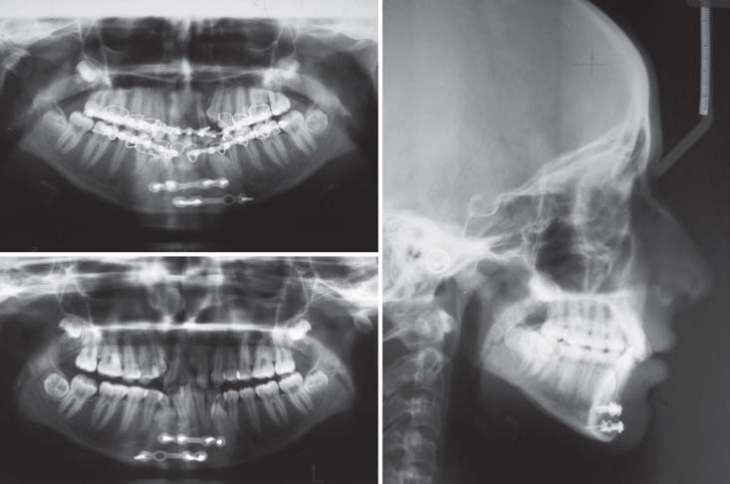
Pretreatment lateral cephalogram and OPG

The treatment was started with arch wiring to hold the fracture segment on the same day. Once the patient is fit for surgery, bone plating was done (Fig. 2).

**Fig. 3:**
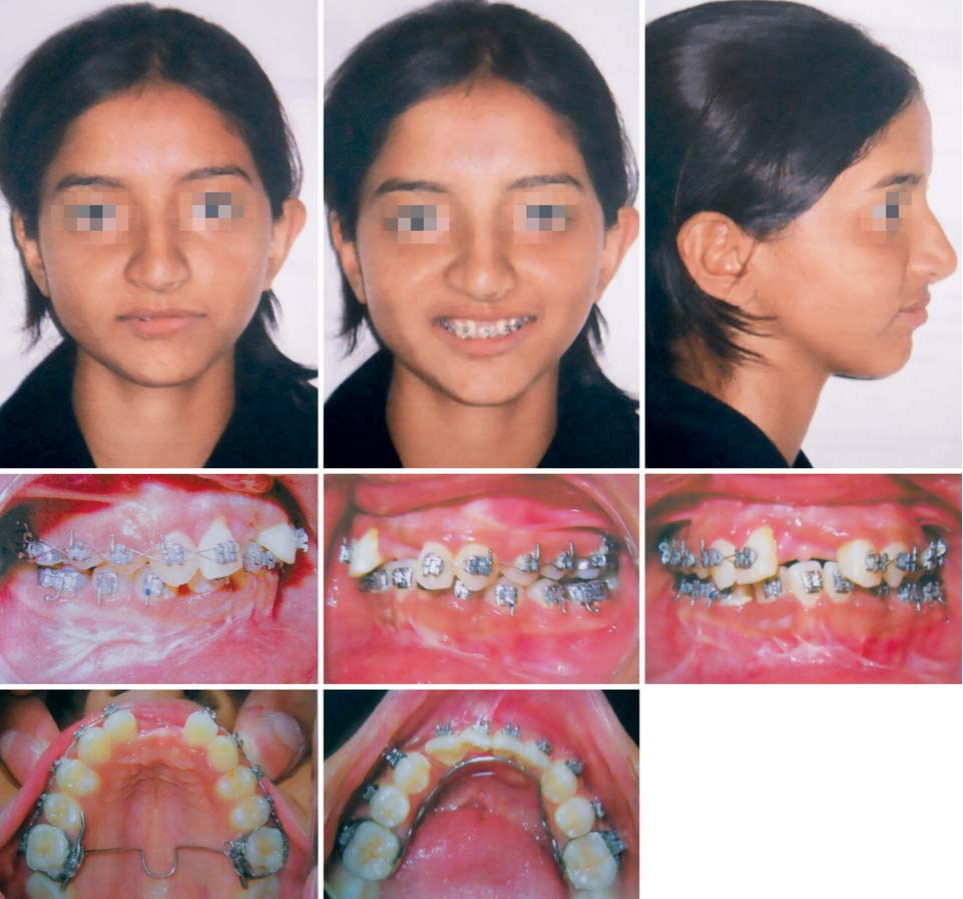
Both the arches were aligned and leveled with fixed mechanotherapy

**Fig. 4:**
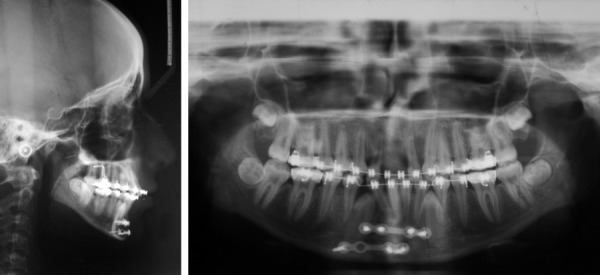
After alignment and leveling lateral cephalogram and OPG

**Fig. 5:**
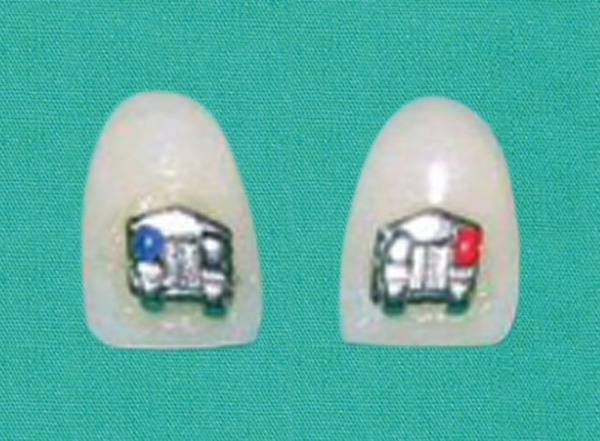
Riding pontics with bracket in place

**Fig. 6:**
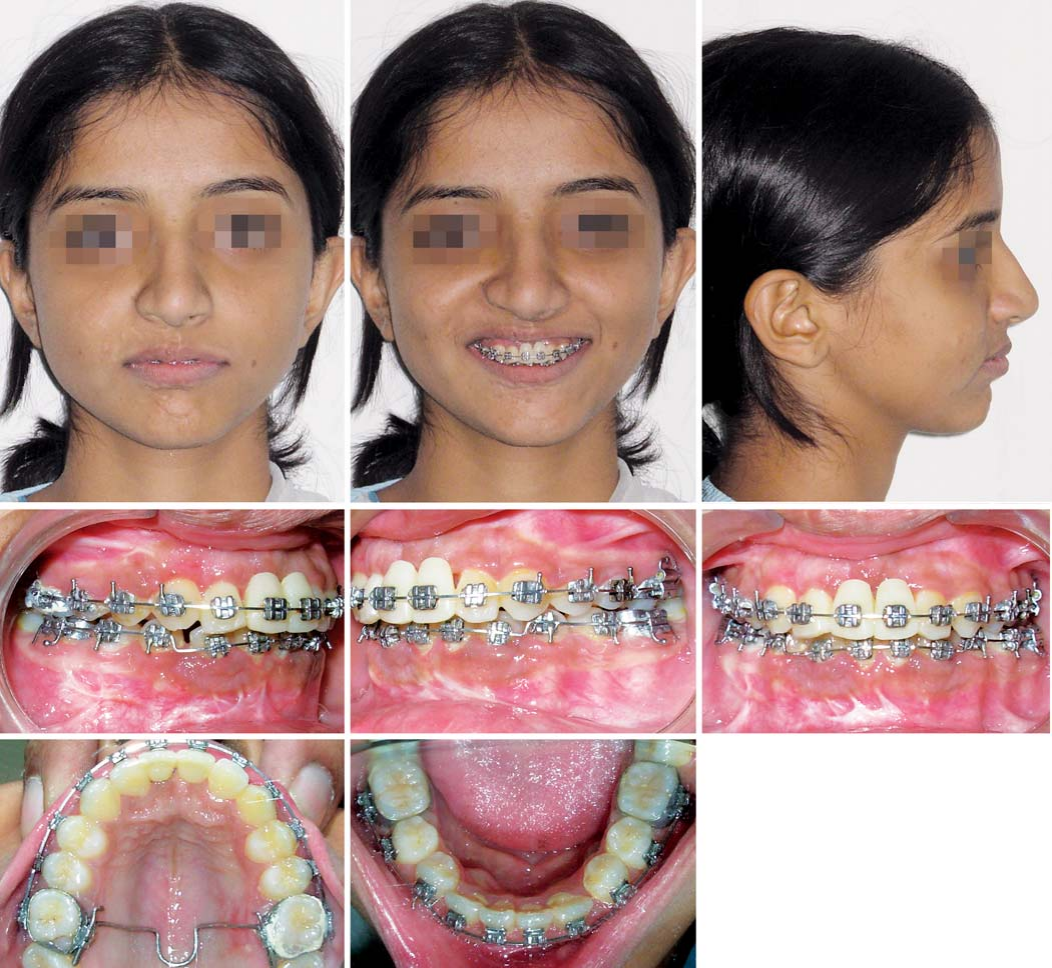
Extraoral and intraoral views with riding pontics in the place

After healing of fracture segment, the malocclusion was treated with fixed mechanotherapy (Roth, 0.022'') (Fig. 3 and 4). Maxillary and mandibular arches were aligned, leveled and space for the central incisors was created. Both mandibular canines were left in bone as they are close to screw and their disimpaction was difficult as they lie at fracture line. Riding pontics were prepared (Fig. 5), trial and adjustment was done, and ligated to the rectangular archwire by stainless steel ligature (Fig. 6). The esthetic and smile of the patient was however improved after ligating riding pontics.

## DISCUSSION

Dentists often encounter patients with congenitally missing or malformed teeth. Treatment options include either orthodontic space maintenance/opening for future restoration or orthodontic space closure. When this situation occurs in the anterior region and the treatment plan calls for maintaining/opening the space, good esthetics during orthodontic treatment can be a concern.^1^ Riding pontics^2^are a better alternative than bonding an acrylic tooth to adjacent teeth.

In past three decades, a major reorientation of orthodontic thinking has occurred regarding adult patients. Most adults seek orthodontic treatment either to improve esthetics or to improve occlusal function. There are several factors for the increased interest by adults in orthodontic treatment including availability of a variety of cosmetic appliances^3^ and an increased desire for treatment of dental mutilation problems using tooth movement and fixed restorations rather than removable prostheses. Edentulous spaces in the anterior region during treatment can be of concern to a patient concern until final the prosthetic replacement. It is extremely important to maintain the esthetics of individuals undergoing fixed orthodontic treatment, especially in patients demanding cosmetic or invisible/lingual appliances. Riding pontics are a good solution in addition to other appliance modifications.^4^

There are few case reports showing the use of riding pontics as space maintainers and esthetic aids in the maxillary anterior region. In cases with the common situation of missing lateral incisors bilaterally,^5^ use of riding pontics are shown to maintain space and a pleasant smile until the end of treatment. In this case with a history of trauma in which a patient had lost his upper incisors and multiple missing teeth were replaced with temporary riding pontics while maintaining esthetics and speech and permitting further space gain. The present article presents information on fabrication and ligation, making usage and placement easy yet effective.

## CONCLUSION

Riding pontic can be considered as an esthetic aid for the orthodontic patients with missing one or more anterior teeth.

It helps to improve patient's esthetic and smile during orthodontic treatment.

What this paper adds:

- Corrective orthodontics
- Diagnosing and treating space problems
- Proper management of space.

Why this paper is important for pediatric dentists:

- Riding pontic can be considered as an esthetic aid for the patients with missing one or more anterior teeth. It helps to improve patient's esthetic and smile during orthodontic treatment.
- This procedure can be easily performed by pedodontist in his office.
- No special skill is required.
- No special armamentarium is required.
